# Experimental infection of chickens as candidate sentinels for West Nile virus.

**DOI:** 10.3201/eid0704.010422

**Published:** 2001

**Authors:** S A Langevin, M Bunning, B Davis, N Komar

**Affiliations:** Centers for Disease Control and Prevention, P.O. Box 2087, Fort Collins, CO 80522, USA.

## Abstract

We evaluated the susceptibility, duration and intensity of viremia, and serologic responses of chickens to West Nile (WN) virus (WNV-NY99) infection by needle, mosquito, or oral inoculation. None of 21 infected chickens developed clinical disease, and all these developed neutralizing antibodies. Although viremias were detectable in all but one chicken, the magnitude (mean peak viremia <10,000 PFU/mL) was deemed insufficient to infect vector mosquitoes. WNV-NY99 was detected in cloacal and/or throat swabs from 13 of these chickens, and direct transmission of WNV-NY99 between chickens occurred once (in 16 trials), from a needle-inoculated bird. Nine chickens that ingested WNV-NY99 failed to become infected. The domestic chickens in this study were susceptible to WN virus infection, developed detectable antibodies, survived infection, and with one exception failed to infect cage mates. These are all considered positive attributes of a sentinel species for WN virus surveillance programs.


### Experimental Infection of Chickens as Candidate Sentinels for West Nile Virus

**Stanley A. Langevin, Michel Bunning, Brent Davis, and Nicholas Komar**
Centers for Disease Control and Prevention, Division of Vector-Borne Infectious Diseases, Fort Collins, Colorado, USA

We evaluated the susceptibility, duration and intensity of viremia, and serologic responses of chickens to West Nile (WN) virus (WNV-NY99) infection by needle, mosquito, or oral inoculation. None of 21 infected chickens developed clinical disease, and all these developed neutralizing antibodies. Although viremias were detectable in all but one chicken, the magnitude (mean peak viremia $<10^4$ PFU/mL) was deemed insufficient to infect vector mosquitoes. WNV-NY99 was detected in cloacal and/or throat swabs from 13 of these chickens, and direct transmission of WNV-NY99 between chickens occurred once (in 16 trials), from a needle-inoculated bird. Nine chickens that ingested WNV-NY99 failed to become infected. The domestic chickens in this study were susceptible to WN virus infection, developed detectable antibodies, survived infection, and with one exception failed to infect cage mates. These are all considered positive attributes of a sentinel species for WN virus surveillance programs.

West Nile (WN) virus is a mosquito-borne zoonosis maintained by birds in Africa, Eurasia, Oceania, and since 1999, North America (1). Since its emergence in recent years, it has become an important public, veterinary, and wildlife health threat. Monitoring the enzootic transmission of WN virus is critical to obtaining an accurate distribution of virus activity and an assessment of risk for human, livestock, and wildlife populations.

Captive sentinel animals, compared to all other arbovirus surveillance systems, provide more precise data on the location and time in which virus transmission has occurred. Chickens are frequently used as sentinels for surveillance of the bird-transmitted arboviral encephalitides. Chickens were equally or more sensitive than other sentinel birds for detecting St. Louis encephalitis virus transmission in Florida and California (2,3). In California, chickens have provided a more sensitive and cost-effective means to early detection of arbovirus activity in comparison to mosquito- and wild bird-based surveillance systems (4). However, chickens have not been evaluated against criteria for a successful sentinel species for WN virus in North America.

A candidate sentinel bird species for the strain of WN virus circulating in North America (WNV-NY99) would be highly susceptible to mosquito-borne infection yet resistant to disease. It must survive infection in order to develop detectable antibodies. Once infected, it should not develop sufficient viremia to infect biting mosquitoes and should not infect either its flock mates (which may skew surveillance results) or its human handlers. In this study, we evaluated domestic chickens against these criteria for a sentinel species for WNV-NY99. In particular, we inoculated chickens by needle, by mosquito, and orally; we measured susceptibility to infection, development of specific antibody, transmission to cage mates, magnitude and duration of viremia, and potential for viral shedding.

Address for correspondence: Nicholas Komar, Centers for Disease Control and Prevention, P.O. Box 2087, Fort Collins, CO 80522, USA; fax: 970-221-6476; e-mail: nkomar@cdc.gov

### Materials and Methods

### Infection of Chickens

Dekalb Delta hens (Hudson Pullet Farm, Fort Lupton, CO) of various ages (17-60 weeks old) were inoculated with WNV-NY99 (source: *Corvus brachyrhynchos* brain 99-41-32, New York State Wildlife Pathology Unit, 1 Vero passage) by needle, mosquito, or oral inoculation. The needle-inoculated birds (n=5) were injected subcutaneously on the breast with 10,000 Vero PFU per 0.05 mL using a 1-cc syringe and a 26-gauge needle. The mosquito-inoculated birds (n=16) were exposed to three to five infected mosquitoes through the mesh-top of a pint-size ice cream container positioned on an exposed region of the hen's breast. The mosquitoes were removed after at least one mosquito had become engorged. For 16 birds, a noninfected cage mate was provided to evaluate contagiousness in the absence of mosquitoes. Oral inoculation was attempted in three groups of three birds by placing 0.2 mL of sterile water containing either 280 PFU WN virus (group 1), 2800 PFU WN virus (group 2), or one infected dead mosquito (group 3) into the gullet, which stimulated the swallow reflex.

All inoculated chickens and their cage mates were bled daily for 7 days postinoculation (dpi). Each day, 0.2 mL of whole blood was withdrawn by jugular or bracheal venipuncture using a 26-gauge, ½-inch subcutaneous needle and added to 0.9 mL of BA-1 diluent (Hanks M-199 salts, 0.05M Tris ph 7.6, 1% bovine serum albumin, 0.35 g/L sodium bicarbonate, 100 units/mL penicillin, 100 mg/mL streptomycin, 1 mg/mL Fungizone). Samples were permitted to coagulate at room temperature for 30 min, centrifuged at 7,000 rpm for 8 min, and frozen at -70°C. Cloacal and throat samples were also taken during the first 7 dpi by using cotton swabs and dipping the infected swabs in 0.5 mL of BA-1 before freezing at -70°C. All inoculated hens were observed twice daily during the first 7 dpi of infection for signs of clinical illness. A final serum sample (0.6 mL of whole blood) was taken at 14 dpi to test for seroconversion by plaque-reduction neutralization test (PRNT) (5). A sample that neutralized the challenge dose of WNV-NY99 by at least 90% was considered positive. Three hens were maintained until 28 dpi to monitor the development of neutralizing antibodies during this period.

### Infection of Mosquitoes

Colonized *Culex tritaeniorhynchus* mosquitoes were infected by intrathoracic inoculation of 700 nL of a suspension containing $10^{8.2}$ per mL WNV-NY99 (source *Cx. pipiens* pool #NY99-6480 collected in New York, 1999, 1 Vero passage, CDC accession no. B82123), and incubated for 7 to 10 days at 16:8 hours light:dark, 28°C, 80% relative humidity, before feeding on chickens. Successful infection of mosquitoes was confirmed by plaque assay of homogenates of whole mosquitoes (after incubation) or saliva extracted from mosquitoes after feeding (6).

### Virus Titration and Identification

The concentration of WN virus infectious particles in fluids (including cloacal swabs, throat swabs, and blood samples) was evaluated by Vero plaque assay (5) of 10-fold serial dilutions. Plaques were counted after 3-5 days of incubation at 37°C, 5% $CO_2$. Plaques from swabs were harvested and identified by neutralization using a standard antiserum available from the Centers for Disease Control and Prevention reference collection in Fort Collins, CO.

### Results

All of the 21 WNV-NY99 parenterally inoculated hens developed neutralizing antibodies and 20 of these had detectable viremia (Table 1). One of 16 in-contact hens had a transient WNV-NY99 viremia of magnitude $10^{2.4}$ PFU/mL on the third day after its cage mate had been injected, and seroconverted. None of nine orally inoculated hens developed WNV-NY99 viremia or antibodies. None of the 46 hens exposed to WNV-NY99 demonstrated overt clinical illness attributable to WN virus.

Three mosquito-infected hens were sampled more frequently (approximately twice per week) after the first week of infection to monitor the pattern of antibody response within 28 dpi (Figure). Neutralizing antibody was detected in one of the three birds as early as 7 dpi (reciprocal 90% neutralization titer = 10), and in all three at 10 dpi (titers = 40, 40, and 80). The titers increased steadily throughout this period, reaching 320, 80, and 160, respectively, by 28 dpi.

We determined the duration and magnitude of WNV-NY99 viremia in the 21 parenterally inoculated hens

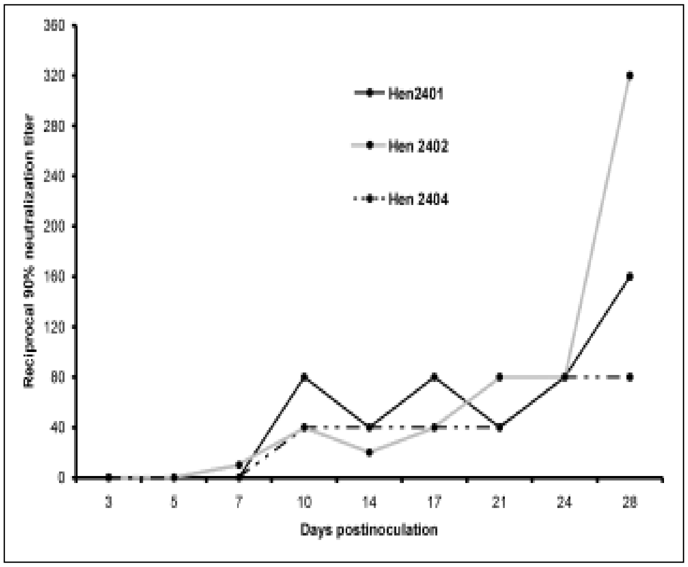

Figure. West Nile virus (WNV)-NY99 neutralizing antibody response in chickens.

Table 1. Viremia in West Nile virus (WNV)-NY99-infected chickens

| Chicken | | Infection | No. mosq. | Day postinoculation | | | | | Cage mate |
|---|---|---|---|---|---|---|---|---|---|
| ID# | Age(wk) | mode | fed | 1 | 2 | 3 | 4 | 5 | infection |
| 1103 | 20 | N | NA | --[a] | 3.7 | 2.7 | -- | -- | - |
| 1108 | 20 | N | NA | -- | 3.8 | 3.1 | -- | -- | + |
| 1110 | 20 | N | NA | 2.0[b] | 5.0 | 3.3 | -- | -- | - |
| 2019 | 20 | N | NA | -- | 2.3 | 3.4 | 2.1 | -- | - |
| 2027 | 20 | N | NA | 1.7 | 3.4 | 2.3 | -- | -- | - |
| 1112 | 17 | M | 4-5 | 3.5 | 3.6 | -- | -- | -- | - |
| 1114 | 17 | M | 4-5 | 3.0 | 3.4 | -- | -- | -- | - |
| 1116 | 17 | M | 4-5 | 3.6 | 3.7 | 2.9 | -- | -- | - |
| 1118 | 17 | M | 4-5 | 3.6 | 3.4 | -- | -- | -- | - |
| 1120 | 17 | M | 4-5 | -- | -- | -- | -- | -- | - |
| 1122 | 17 | M | 4-5 | -- | -- | 3.0 | 2.8 | -- | - |
| 2401 | 17 | M | 1 | -- | 1.7 | 3.4 | 2.3 | -- | NT |
| 2402 | 17 | M | 2 | -- | 2.7 | 3.1 | -- | -- | NT |
| 2404 | 17 | M | 1 | -- | 2.4 | 2.2 | -- | -- | NT |
| 2595 | 17 | M | 1 | -- | 2.6 | 3.5 | 2.2 | -- | NT |
| 2596 | 17 | M | 1 | 2.4 | 3.5 | -- | -- | -- | NT |
| 1124 | 60 | M | 1 | 2.9 | 3.6 | 2.0 | -- | -- | - |
| 1126 | 60 | M | 1 | -- | 4.0 | 3.4 | -- | -- | - |
| 1128 | 60 | M | 1 | -- | 3.6 | 2.9 | -- | -- | - |
| 1132 | 60 | M | 1 | -- | 3.9 | 2.8 | -- | -- | - |
| 1134 | 60 | M | 1 | 4.1 | 3.9 | -- | -- | -- | - |

[a]Threshold of detection is 50 PFU/mL serum.
[b]$\log_{10}$ Vero PFU/mL serum.
N = needle; NA = not applicable; M = mosquito; NT = not tested.

(Table 1). All five hens inoculated by needle had detectable viremias that endured 2 to 3 days with mean peak viremia of $10^{3.9}$ PFU/mL (range $10^{3.4}$-$10^{5.0}$). Of the 16 hens inoculated by mosquito, 15 had detectable viremias that endured 2 to 3 days with mean peak viremia of $10^{3.4}$ (max $10^{4.1}$). No virus was detected in blood samples collected 6 and 7 dpi (data not shown).

Cloacal shedding of WNV-NY99 was observed in 12 of 21 (57%) parenterally inoculated hens (Table 2). All 5 of the needle-inoculated birds and all 5 of the 60-week-old mosquito-inoculated birds shed, whereas only 2 of 11 (18%) 17-week-old mosquito-inoculated birds shed. Positive cloacal swabs were observed 2-6 dpi. Peak cloacal swab positivity was 3-5 dpi. Shedding in oral exudates was observed in two of six 17-week-old hens. In these six birds, the number of plaques detected from throat swabs was generally less than that from cloacal swabs (Table 2). Viruses detected in swabs were identified as WN virus by PRNT and were reisolated from a subset of the positive swabs for confirmation of results. To evaluate the viability and stability of WNV-NY99 in fecal material outside the host, fecal urates of chickens were mixed with 100 PFU WNV-NY99. No negative effect of the fecal material was observed when compared with BA-1 diluent. However, viability was reduced by 99% after 24 hours at ambient temperature (data not shown).

### Discussion

This study evaluated WNV-NY99 sentinel criteria for chickens by monitoring their response to experimental infection in captivity. We report for the first time quantitative data about WNV-NY99 viremias in chickens inoculated by mosquito bite. Turell (7,8) reported that chicks were infected with WN virus by mosquito bite, but data from these evaluations were not presented. The response of several bird species (including chickens, turkeys, and geese) to needle inoculation of this North American strain of WN virus has been documented (9-11). However, mosquito inoculation has been shown to elicit a different response to infection compared with needle inoculation in several vertebrate-virus systems (12,13).

Three central criteria for an arbovirus sentinel bird are susceptibility to infection, development of detectable antibodies, and survival. Birds that do not survive infection may be lost to surveillance programs designed to detect antibodies as a marker for infection. We found that all the chickens inoculated parenterally in our study, as in other WN virus infection studies in chickens (14,15), became infected, and survived to develop detectable neutralizing antibodies. Evaluation of alternative serodiagnostic assays for immunoglobulin M and hemagglutination-inhibiting antibodies are under way.

Birds used as sentinels for arbovirus surveillance should not contribute to the local arbovirus transmission cycle if they become infected. Detectable viremia in mature chickens (>3 weeks) is unusual for WN virus strains that have been studied previously (14,15), although young chicks do develop viremia $>10^5$ PF/mL (14, 7). Senne et al. (9) reported that WNV-NY99 needle-inoculated 7-week-old hens had viremia sufficient to infect mosquitoes, based on data from an experimental infection study of an African mosquito, *Cx. univittatus*, using an African strain of WN virus (cited in 16). However, new data do not support this statement. A study of vector competence of *Cx. pipiens* collected in New York and infected with WNV-NY99 suggests that the maximum viremia that we observed in the needle-inoculated hens ($10^5$ PFU/mL) is sufficient to infect about 17% of these mosquitoes; 2% will be able to transmit the virus in a subsequent bloodmeal (7). The maximum viremia detected in mosquito-inoculated hens reached $10^{4.1}$ and is probably well below the level required to maintain the *Cx. pipiens* transmission cycle. Although other species of mosquitoes may have lower thresholds of infection, *Cx. pipiens* is recognized as the important vector in the avian transmission cycle in the northeastern United States (17). Thus, our data imply that chickens are incompetent to retransmit WNV-NY99 to *Cx. pipiens* in New York. However,

Table 2. West Nile virus (WNV)-NY99 PFU in 0.5 mL cloacal or throat swabs of chickens

| Chicken | | Infection | No. mosq. | Day postinoculation | | | | | | |
|---|---|---|---|---|---|---|---|---|---|---|
| ID# | Age (wk) | mode | fed | 1 | 2 | 3 | 4 | 5 | 6 | 7 |
| 1103 | 20 | N | NA | NT | NT | 23[a] | 0 | NT | 5 | 0 |
| 1108 | 20 | N | NA | NT | NT | 200 | 0 | NT | 0 | 0 |
| 1110 | 20 | N | NA | NT | NT | 28 | 95 | NT | 3 | 0 |
| 2019 | 20 | N | NA | NT | NT | 3 | 8 | NT | 0 | 0 |
| 2027 | 20 | N | NA | NT | NT | 10 | 5 | NT | 0 | 0 |
| 1112 | 17 | M | 4-5 | 0/3[b] | 4/0 | 6/3 | 23/3 | 8/0 | 0/0 | 0/0 |
| 1114 | 17 | M | 4-5 | 0/0 | 0/0 | 0/0 | 0/0 | 0/0 | 0/0 | 0/0 |
| 1116 | 17 | M | 4-5 | 0/0 | 0/0 | 0/0 | 0/3 | 0/0 | 0/0 | 0/0 |
| 1118 | 17 | M | 4-5 | 0/0 | 0/0 | 4/0 | 1/0 | 0/0 | 3/0 | 0/0 |
| 1120 | 17 | M | 4-5 | 0/0 | 0/0 | 0/0 | 0/0 | 0/0 | 0/0 | 0/0 |
| 1122 | 17 | M | 4-5 | 0/0 | 0/0 | 0/0 | 0/0 | 0/0 | 0/0 | 0/0 |
| 2401 | 17 | M | 1 | 0 | 0 | 0 | 0 | 0 | 0 | 0 |
| 2402 | 17 | M | 2 | 0 | 0 | 0 | 0 | 0 | 0 | 0 |
| 2404 | 17 | M | 1 | 0 | 0 | 0 | 0 | 0 | 0 | 0 |
| 2595 | 17 | M | 1 | 0 | 0 | 0 | 0 | 0 | 0 | 0 |
| 2596 | 17 | M | 1 | 0 | 0 | 0 | 0 | 0 | 0 | 0 |
| 1124 | 60 | M | 1 | 0 | 0 | 0 | 0 | 5 | NT | NT |
| 1126 | 60 | M | 1 | 0 | 0 | 6 | 5 | 0 | NT | NT |
| 1128 | 60 | M | 1 | 0 | 0 | 0 | 0 | 1 | NT | NT |
| 1132 | 60 | M | 1 | 0 | 0 | 3 | 11 | 18 | NT | NT |
| 1134 | 60 | M | 1 | 0 | 3 | 9 | 24 | 3 | NT | NT |

[a]Data presented are from cloacal swabs unless otherwise indicated.
[b]Cloacal swab/nasopharyngeal swab.
N = needle; NA = not applicable; NT = not tested; M = mosquito.

we recognize that conditions may exist in which vector mosquitoes, including strains of *Cx. pipiens*, could theoretically have lower transmission thresholds that permit them to acquire WNV-NY99 infection from mature chickens.

Birds used as sentinels for arbovirus surveillance should not spread arbovirus infections directly to flock mates, because a finding of birds that are seropositive as a result of direct transmission (in the absence of mosquito vectors) would lead to misinterpretation of the true risk for mosquito-borne transmission. In our study, we observed one such transmission event (out of 16 trials). This transmission originated from a needle-inoculated hen. Experimental direct transmission (from needle-inoculated birds) has been observed with other WNV-NY99-infected species, including domestic goslings (11) and American Crows (R.G. McLean, pers. comm.), but not chicken pullets and turkey poults (9,10). The importance of direct transmission of WNV-NY99 among birds in nature remains unknown.

The means by which WNV-NY99 direct transmission among birds occurs may include inhalation of infectious aerosols due to viral shedding in bodily fluids such as fecal material and saliva, ingestion of contaminated food, or contact with viremic blood. The possibility of oral ingestion of WNV-NY99 was tested in nine chickens with negative results. We did, however, document the presence of infectious WNV-NY99 in oral exudates and feces. WNV-NY99 has been reported previously in cloacal swabs of needle-inoculated chicken pullets and turkey poults (9,10) but not goslings (11), and in oropharyngeal swabs of turkeys and goslings (10,11). We observed that the quantity of virus collected in swabs was relatively low (not exceeding 200 infectious virus particles in our preliminary evaluation) and that stability of WNV-NY99 in avian fecal material outside the host was reduced dramatically after 24 hours, suggesting that risk of transmission from infected feces decreases as the time outside the host increases. Because WN virus infection in humans exposed to viral shedding in birds has not been documented, the actual risk is unknown and can be reduced through proper recommended animal-handling techniques, such as the use of disposable gloves and HEPA-filtered masks.

Chickens should be evaluated as sentinels for detecting and monitoring enzootic WN virus transmission. Chickens have been used extensively for surveillance of Kunjin virus (a subtype of WN virus) in Australia (R. Russell, pers. comm.). Pre-existing flocks of domestic chickens were naturally exposed to WN virus in Bucharest in 1996 (37% seropositive) (18), New York City in 1999 (63%) (19), and eastern Suffolk County, NY, in 1999 (30%, S. Campbell, pers. comm.). Thus, based on these data, chickens would seem to be strong candidates for use as sentinels for WN virus.

In summary, we present the first experimental infection study of WNV-NY99 in chickens in which mosquito and oral transmission routes are evaluated. We found that WNV-NY99 viremia in chickens is probably insufficient to infect the primary epiornitic vector, *Cx. pipiens*. The observation of transmission to a hen in contact with a needle-inoculated WNV-NY99-infected hen requires further study on the risk of direct transmission among chickens and to their handlers by contaminated bodily fluids. This experimental infection study provides data that, in part, justify chickens as candidates for WN virus sentinels in North America.

### Acknowledgments

We thank Robert Craven, John Roehrig, Lyle Petersen, Duane Gubler, and two anonymous reviewers for critical review of the manuscript. Ezra Jones and Jameson Stokes provided technical assistance.

Mr. Langevin is a fellow in training at the Centers for Disease Control and Prevention, Arbovirus Diseases Branch, Fort Collins, Colorado. His major research interests are zoonotic diseases.

### References

1. Komar N. West Nile viral encephalitis. Rev Sci Tech 2000;19:166-76.
2. Reisen WK, Hardy J, Presser S. Evaluation of domestic pigeons as sentinels for detecting arbovirus activity in southern California. Am J Trop Med Hyg 1992;46:69-79.
3. Morris CD, Baker WG, Stark L, Burgess J, Lewis AL. Comparison of chickens and pheasants as sentinels for eastern equine encephalitis and St. Louis encephalitis viruses in Florida. J Am Mosq Control Assoc 1994;10:545-8.
4. Reisen WK, Presser SB, Lin J, Enge B, Hardy JL, Emmons RW. Viremia and serological responses in adult chickens infected with western equine encephalomyelitis and St. Louis encephalitis viruses. J Am Mosq Control Assoc 1994;10:549-55.
5. Beaty BJ, Calisher CH, Shope RE. Arboviruses. In: Schmidt NJ, Emmons RW, editors. Diagnostic procedures for viral, rickettsial and chlamydial infections. 6th ed. Washington: American Public Health Association; 1989. p. 797-855.
6. Hurlbut HS. Mosquito salivation and virus transmission. Am J Trop Med Hyg 1966;15:989-93.
7. Turell MJ, O'Guinn M, Oliver J. Potential for New York mosquitoes to transmit West Nile virus. Am J Trop Med Hyg 2000;62:413-4.
8. Turell MJ, O'Guinn ML, Dohm DJ, Jones JW. Vector competence of North American mosquitoes (Diptera: Culicidae) for West Nile virus. J Med Entomol 2001;38:130-4.
9. Senne DA, Pedersen JC, Hutto DL, Taylor WD, Schmitt BJ, Panigrahy B. Pathogenicity of West Nile virus in chickens. Avian Dis 2000;44:642-9.
10. Swayne DE, Beck JR, Zaki S. Pathogenicity of West Nile virus for turkeys. Avian Dis 2000;44:932-7.
11. Swayne DE, Beck JR, Smith C, Shieh W, Zaki S. Fatal encephalitis and myocarditis in young domestic geese (*Anser anser domesticus*) caused by West Nile virus. Emerg Infect Dis 2001;7:751-3.
12. Osorio JE, Godsey MS, Defoliart GR, Yuill TM. La Crosse viremias in white-tailed deer and chipmunks exposed by injection or mosquito bite. Am J Trop Med Hyg 1996;54:338-42.
13. Edwards JF, Higgs S, Beaty BJ. Mosquito feeding-induced enhancement of Cache Valley virus (Bunyaviridae) infection in mice. J Med Entomol 1998;35:261-5.
14. Taylor RM, Work TH, Hurlbut HS, Rizk F. A study of the ecology of West Nile virus in Egypt. Am J Trop Med Hyg 1956;5:579-620.
15. Parks JJ, Ganaway JR, Price WH. Studies on immunologic overlap among certain arthropod-borne viruses III. A laboratory analysis of three strains of West Nile virus which have been studied in human cancer patients. Am J Hyg 1958;68:106-19.
16. McIntosh BM, Jupp PG. Infections in sentinel pigeons by Sindbis and West Nile viruses in South Africa, with observations on *Culex (Culex) univittatus* (Diptera: Culicidae) attracted to these birds. J Med Entomol 1979;16:234-9.
17. Nasci RS, White DJ, Stirling H, Oliver J, Daniels TJ, Falco RC, et al. West Nile virus isolates from mosquitoes in New York and New Jersey, 1999. Emerg Infect Dis 2001;7:626-30.
18. Savage HM, Ceianu C, Nicolescu G, Karabatsos N, Lanciotti R, Vladimirescu A, et al. Entomologic and avian investigations of an epidemic of West Nile fever in Romania in 1996, with serologic and molecular characterization of a virus isolate from mosquitoes. Am J Trop Med Hyg 1999;61:600-11.
19. Komar N, Panella NA, Burns JE, Dusza SW, Mascarenhas TM, Talbot T. Serologic evidence for West Nile virus infection in birds in the New York City vicinity during an outbreak in 1999. Emerg Infect Dis 2001;7:621-5.
